# Transfusion Transmitted Hepatitis: Where Do We Stand Now? A One Center Study in Upper Egypt

**DOI:** 10.5812/hepatmon.852

**Published:** 2012-04-30

**Authors:** Amel Abdel Magied El-Faramawy, Omnia Fathy El-Rashidy, Perihan Hamdy Tawfik, Galal Helmy Hussein

**Affiliations:** 1 The Pediatrics Department, Faculty of Medicine, Ain Shams University, Cairo, Egypt; 2 The Clinical Pathology Department, Faculty of Medicine, Ain Shams, University, Cairo, Egypt

**Keywords:** Child, Hepatitis, Transfusion, Egypt

## Abstract

**Background:**

Despite progress made in the prevention of transfusion-transmitted infections (TTI) over the last few years, they continue to be a problem in many parts of the world, particularly in multitransfused patients.

**Objectives:**

The aim of this study was to estimate the prevalence of hepatitis B virus (HBV), hepatitis C virus (HCV), and to evaluate the screening and vaccination program among our cohort of multitransfused children from Qena, Upper Egypt.

**Patients and Methods:**

One-hundred children suffering from diseases requiring repeated blood transfusions were included in the study. They were classified into group 1, which included 67 children with thalassemia, and group 2, which included 33 children with hemophilia. Screening for hepatitis B surface antigen, hepatitis B surface antibody, hepatitis B core antibody and antibody to HCV was done using a second-generation enzyme-linked immunosorbent assay technique.

**Results:**

Only 12% of all patients were either acutely or chronically infected with HBV. 46% were immune due to previous vaccination, whereas 39% of patients were not protected from HBV infection. HCV antibodies were positive in 45% of cases. Seventy-eight patients had a complete hepatitis B vaccination in the form of three doses as documented by birth certificate. Thirty-six patients mentioned history suggestive of hepatitis. The prevalence of the studied hepatitis markers was similar in both the thalassemia and hemophilia groups of children.

**Conclusions:**

Transfusion-transmitted hepatitis is still a major problem for multitransfused children in Egypt. More effort is required to reduce the infection rate through proper screening of blood and blood products, strict emphasis on receiving the vaccine, regular follow-up for those children with a hepatitis B antibody titer, and providing booster doses for those in need.

## 1. Background

Regular blood transfusions improve the overall survival of multitransfused children but despite the progress made in preventing transfusion-transmitted infections (TTIs) over the last few years, TTIs continue to be a problem in many parts of the world [1]. Blood transfusion is the main risk factor for transmitting viral hepatitis, particularly in patients with hematological diseases [2].The TTI problem is directly proportional to the prevalence of infection in the blood donor community [3]. Patients with thalassemia commonly receive transfusions and, thus, are exposed to transfusion-associated infections. Among these infections, hepatitis B and hepatitis C are the most common [4]. Hepatitis B is an important infection in patients with thalassemia and prevention by vaccination is necessary. The immunological response to hepatitis B vaccine in polytransfused patients with thalassemia requires additional research attention [5]. Although the risk of post-transfusion hepatitis C virus (HCV) infection dropped significantly after the national screening of blood in 1993, more than 20% of children who were multitransfused after that date were HCV-RNA positive [6]. Monitoring infectivity markers in multitransfused patients is an important indicator of the efficiency and quality of testing in blood centers [7].

## 2. Objectives

The objective of the study was to estimate the prevalence of hepatitis B virus (HBV) and HCV antibodies among multitransfused children registered at the blood bank of Red Crescent Hospital in Qena (Upper Egypt) to update the prevalence of HBV and HCV infections among multitransfused children and to evaluate the impact of serological screening and the vaccination program on the prevalence of those infections.

## 3. Patients and Methods

This cross-sectional study included 100 children attending the children’s blood bank established to serve children at Red Crescent Hospital. The children were suffering from diseases that require repeated blood transfusions. This study was conducted according to the recommendations of the Ethics Committee of Children’s Hospital, Ain Shams University. Informed consent was obtained from the children’s guardians. Among the 100 included patients, 67 had thalassemia and constituted group 1, 33 patients were hemophiliacs and constituted group 2. The groups were subjected to a complete history stressing the duration of transfusion therapy, blood source (Red Crescent blood bank or multiple centers), frequency of transfusion, history of vaccination against hepatitis B, and a history suggestive of hepatitis. A thorough clinical examination was performed, emphasizing the presence or absence of jaundice and size of the liver and spleen. Five cc of venous blood was collected from each patient during regular follow-up using a sterile plastic syringe. One cc was mixed with EDTA and used for a complete blood count (CBC). The blood samples were centrifuged for 10 minutes. The remaining serum was used to measure aspartate transaminase (AST), alanine transaminase (ALT), and total and direct bilirubin levels. The remaining serum was placed in sterile covered storage tubes and stored at −30°C until collection of all samples. Screening for the hepatitis B surface antigen (HBsAg), hepatitis B surface antibody, and hepatitis B core antibody (HBc Ab) was conducted using a second-generation enzyme-linked immunosorbent assay (ELISA) technique with commercial kits provided by ADALTIS (Guidonia di Montecelio, Rome, Italy) Screening for antibody to HCV (anti-HCV) was performed using a second generation ELISA technique with commercial kits provided by Diasorin (Saluggia (Vercelli), Italy).

### 3.1. Statistical Analysis

Descriptive and analytical statistics were performed using the Excel 2003 software package (Microsoft Inc., Redmond, WA, USA). Data are presented as mean ± SD and range. Categorical data are presented as numbers and percentages. Comparisons between the groups were made using the Mann-Whitney U-test for non-parametric data (z-values). Associations between categorical parameters were performed with the chi-square test. Values of P < 0.05 were considered significant.

## 4. Results

The patients included in the study ranged in age from 4–15 years with a mean of 8.29 ± 3.16 years. There were 68 (68%) males and 32 (32%) females. Their disease duration ranged from 2 to 13 years with a mean of 6.45 ± 3.11 years. Children with thalassemia were treated with transfusions of packed red blood cells (RBCs), chelation therapy, and a splenectomy, if indicated. Children with hemophilia received either fresh frozen plasma or cryoprecipitate. The transfused blood or blood product units (packed RBCs unit = 300 ml, plasma unit = 250 ml, and cryoprecipitate unit = 15 ml) ranged from 16 to 149 units with a mean of 66.90 ± 33.69 units through the entire disease duration. Forty-four patients received blood or blood products from the Red Crescent blood bank at Qena, and 56 patients (56%) received blood from multiple centers. Seventy-eight patients had complete hepatitis B vaccinations in the form of three doses as documented by birth certificate, and 22 patients (22%) had incomplete vaccination of hepatitis B (missing one or two doses). Thirty-six patients mentioned a history suggestive of hepatitis. Fifty-one of the patients were on chelation therapy due to high serum ferritin levels (>1000 ng/dl), and 23 patients (23%) were splenectomized, as they were all diagnosed with thalassemia. As shown in Table 1, 12% of all patients were positive for HBsAg and were either acutely (5%) or chronically (7%) infected with hepatitis B. In total, 46 patients were immune due to vaccination and three were immune due to previous infection, whereas 39% of patients were not protected from HBV infection. HCV antibodies were positive in 45 cases and coinfection by hepatitis B and C was present in 12. Eight males and four females were positive for HBsAg. Their mean age was 11.2 ± 3.3 years with a mean disease duration of 8.5 ± 3 years. Seven/12 were not completely vaccinated. Thirty-two males and 13 females had positive HCV antibodies. Their mean age was 7.7 ± 2.8 years with a mean disease duration of 3.5 ± 1.9 years. Children positive for HBsAg were significantly older than those who were positive for HCV (P < 0.001). They also had a longer disease duration (P < 0.001) and received more blood or blood products units (P < 0.05).

No relationship was found with the blood source.

**Table 1:** Hepatitis Markers For the Studied Cases

**Hepatitis Markers**	**Cases, No. (%) (n=100)**	**Significance**
HBs Ag: negative	46 (46)	Indicating Immunity because of hepatitis B vaccination
HBs Ab: positive		
HBc Ab: negative		
HBs Ag: positive	7(7)	Indicating chronic infection
HBs Ab: positive		
HBc Ab: negative		
HBs Ag: positive	5(5)	Indicating that they are acutely infected
HBs Ab: negative		
HBc Ab: positive		
HBs Ag: negative	3(3)	
HBs Ab: positive		Immune because of natural infection
HBc Ab: positive		
HBs Ag: negative	39(39)	
HBs Ab: negative		Susceptible (not immune) to HBV infection
HBc Ab: negative		
HCV Ab: positive	45(45)	Indicating recent or past infection with HCV
Positive for both HBs Ag and HCV Ab	12(12)	have co infection

Patients were classified into two groups. Group 1 included 67 patients with ß-thalassemia and group 2 included 33 patients with hemophilia. Table 2 shows a comparison of the studied parameters between the two groups.

Patients in group 2 were significantly older and had a prolonged disease duration compared to those in group 1. Significantly more blood or blood products units were received by group 2 patients compared to those in group 1 (P < 0.001). AST (IU/L) and direct bilirubin (mg/dl) were significantly higher in group 2 than those in group 1, whereas significantly higher total bilirubin (mg/dl) (P < 0.05) and indirect bilirubin (mg/dl) (P < 0.001) levels were observed in group 1 compared to those in group 2 (Table 2).

**Table 2:** Clinical and Laboratory Studies of the Two Groups

	**Group 1**	**Group 2 **	**P value**
Age, y, mean ± SD	7.64 ± 2.93	8.94 ± 3.29	< 0.05
Disease duration, y, mean ± SD	5.62 ± 2.63	7.27 ± 3.35	< 0.05
Received blood, units, mean ± SD	50.94 ± 23.57	82.86 ± 34.92	< 0.001
AST, IU/L, mean ± SD	32.14 ± 21.11	47.32 ± 24.40	< 0.05
ALT, IU/L, mean ± SD	35.84 ± 19.09	47.70 ± 29.42	> 0.05
T bilirubin, mg/dL, mean ± SD	2.90 ± 1.16	2.26 ± 0.91	< 0.05
D bilirubin, mg/dL, mean ± SD	1.06 ± 0.74	1.42 ± 0.76	< 0.05
Indirect bilirubin, mg/dL, mean ± SD	1.84 ± 0.95	0.84 ± 0.57	< 0.001
Source of transfused blood, No. (%)			> 0.05
RCB a only	31 (46)	13 (39)	
Multiple sources	36 (54)	20 (61)	
History of HB Vaccination, No. (%)			> 0.05
Incomplete	12 (18)	9 (27)	
Complete	55 (82)	24 (73)	
History suggestive of hepatitis			> 0.05
Negative	42 (63)	22 (67)	
Positive	25 (37)	11 (33)	

^a^ Abbreviations: RCB, Red Crescent blood bank

The prevalence of the hepatitis markers was similar in the thalassemia and hemophilia groups of children (Table 3).

**Table 3:** Viral Markers in the Two Groups

**Hepatitis Markers**	**Interpretation**	**Group 1, No. (%) (n = 67),**	**Group 2, No. (%) (n = 33)**	**P value **
HBs Ag: Negative	Immune due to vaccination against HBV	31 (46)	15 (46)	> 0.05
HBs Ab: Positive				
HBc Ab: Negative				
HBs Ag: Positive	Chronically infected	3 (4)	4 (12)	> 0.05
HBs Ab: Positive				
HBc Ab: Negative				
HBs Ag: Positive	Acutely infected	3 (4)	2 (6)	> 0.05
HBs Ab: Negative				
HBc Ab: Positive				
HBs Ag: Negative	Immune due to natural infection	2 (3)	1 (3)	> 0.05
HBs Ab: Positive				
HBc Ab: Positive				
HBs Ag: Negative	Not immune, at risk & need vaccination	28 (42)	11 (33)	> 0.05
HBs Ab: Negative				
HBc Ab: Negative				
HCV Ab: Positive	Recent or past infection with HCV.	32 (48)	13 (39)	> 0.05
HBs Ag and HCV Ab	Coinfection	7 (10)	5 (15)	> 0.05

## 5. Discussion

In 1992, Egypt started a universal immunization program in infants. The schedule adopted by the Egyptian Ministry of Health was three doses of yeast-recombinant hepatitis B vaccine administered to all infants at 2, 4, and 6 months to coincide with other compulsory vaccines [8]. However, complete hepatitis B vaccination in our study (as documented by birth certificate) was achieved in 78 patients, whereas 22 patients had incomplete vaccination (missed one or two doses). This result suggests that the vaccination schedule requires additional effort to ensure that the entire population has strictly completed their vaccinations, particularly in the high-risk groups of patients. We also found that among all polytransfused children in our study that one-third of those who completed their vaccinations were not protected. This could be explained by the fact that a certain percentage of those who received vaccinations will be non-responsive or hyporesponsive to the HBV vaccine; this is particularly true for patients with chronic liver disease [9], renal disease, or patients undergoing hemodialysis, as these patients lose hepatitis B immunity after natural infection or vaccination [10]. The prevalence of HbsAg in polytransfused patients was high before the era of vaccination, as shown in different studies, and it varies in different places according to prevalence in the community. Amarapurkar et al. [11] studied children with thalassemia for HBsAg in India in 1992. The results were positive in 45% of their patients. In 1993, a study among Egyptian children with thalassemia showed that HBsAg positivity was 16.48% [12]. After establishing a vaccination program against hepatitis B, there was a notable impact on the prevalence of hepatitis B infection rate. Al-Shayyab et al. [13] showed HBsAg seropositivity in 3.5% of patients with hereditary hemolytic anemia in a study conducted in Jordan in 2001i. Irshad et al. [14] reported that 18% of children with thalassemia were positive for HBsAg in India in 2002. In 2005, Khakhkhar and Joshi [15] conducted a study on children with thalassemia also in India and found that only 6.6% were positive for HBsAg. A very low prevalence of positive HBsAg was found in multi-transfused patients with β-thalassemia major or intermedia and sickle cell anemia in a 2006 Turkish study by Ocak et al. [16], as only 0.75% were HBsAg positive. Among 50 patients with thalassemia, hepatitis B virus infection occurred in one patient (2%) in Cairo in 2009 [17].

The decrease in the prevalence of hepatitis B infection reflects the efficacy of the vaccination program and effective screening of blood and blood products. Despite the decline in the prevalence of HBsAg among Egyptian polytransfused children over the past 20 years, it is still high compared to other geographical areas, and this could be attributed to the high infection rates among those children despite vaccination against HBV, as they might not have responded adequately to vaccination or may not be fully vaccinated. Post-vaccination infections may be a result of viruses harboring surface (S)-gene mutations in a region critical for antibody reactivity to the hepatitis B surface antigen (anti-HBs). Furthermore, the burden of infection in the community plays a role, as Egypt has intermediate levels of HBV infection (2–7% chronic infection) [18]. Although blood is screened for HBV in blood banks in Egypt, blood-related HBV transmission may occur, because screening in most blood banks is performed only for HBsAg but not for core antibodies or ALT. El Zayadi et al. [19] called for implementing the anti-HBc test as a routine assay, which would certainly eliminate possible HBV-infected blood units, as rejection of these units would be beneficial to decrease the risk of HBV transmission, particularly in immunocompromised recipients. Several published studies have reported the prevalence of HCV among polytransfused patients in different areas over the world. Khalifa et al. [20] reported HCV prevalence of 85% in Egypt in 1993, and the prevalence of HCV in Egypt in 1997 was 69% among polytransfused children and 55% among blood donors [21]. The prevalence of HCV positivity in New Delhi in 1998 was 57% [22]. In Denmark, 56% of polytransfused patients were HCV positive in 2002 [23], whereas in Iran Ansar et al. [24] found HCV antibodies in 63.8% of their polytransfused patients. The reported prevalence of hepatitis C antibodies in Italy was 40.7% in 2002 [25]. Darwish et al. [26] studied the geographic distribution of HCV sero-positivity in patients with thalassemia in Egypt and found that 46.2% in the Delta region were HCV positive, which is the highest prevalence in Egypt compared to 38.6% in Big Cairo and 37.8% in Upper Egypt. In Iran in 2008, 16.9% of children with thalassemia, were anti-HCV positive [27], whereas in Khuzestan Province, southwest Iran, 28.1% of patients with thalassemia were HCV positive in 2009 [28]. However, the HCV prevalence was 41.7% in a study by Hussain et al. [29] conducted in Pakistan in 2008. In the Middle East, the prevalence of HCV among patients with thalassemia has been remarkably stable at 40–70% from 1993 to 2001. The highest prevalence has been reported in Egypt at 75.6% and the lowest at 40% in Bahrain. In Jordan, HCV prevalence was 40.5% [13], whereas in Lebanon, 14% of patients were positive for HCV [25]. In Saudi Arabia, a prevalence of 26% was reported in 2002 [30], while in 2011, 1.1% of multitransfused children had hepatitis B surface antigen, 3.5% were anti-HCV-positive, and none were anti-HIV-positive [31]. The current and future burden of diseases caused by viral hepatitis in Egypt is significant. It is not an exaggeration to say that viral hepatitis (particularly HCV) is currently and will remain Egypt’s most pressing public health issue for some time. HBV accounts for 10–30% of chronic liver disease, and there is a large occult reservoir of HCV causing chronic liver disease [32]. Omar et al. [33] concluded that despite the decrease in HCV antibody prevalence from 71% to 51.7% in 1995, HCV infection still represents a major health problem for patients with thalassemia, which requires more attention and effort. The high prevalence of HCV in our study was expected, because the prevalence of HCV among the Egyptian population is one of the highest in the world. The screening for HCV by ELISA, which is used in Egypt, is not very accurate, and many countries are using better techniques for screening donated blood for viruses by recombinant immunoblot assay and polymerase chain reaction techniques. Moreover, the donor could be in a window period during which infection may not be detected serologically [34]. In our study, 39% of patients with hemophilia were HCV positive, which is lower than what was found in a study conducted in Brazil, whereas 44.6% of the hemophilic population was positive [35], but higher than the results found by Asif et al. [36], in Pakistan, where 36% of patients with hemophilia A and B tested positive for the hepatitis C antibody. Rezvan et al . [37] reported that the prevalence of anti-HCV in patients with hemophilia is significantly higher than that in other multitransfused patients. They reported that patients with hemophilia are more susceptible to TTIs, as their treatment is dependent on the use of large-pool coagulation factor concentrates as well as fresh frozen plasma and cryoprecipitate. Although HCV transmission by blood products is prevented by viral inactivation steps now used in the production of plasma-derived factor concentrates, transmission remains a problem in many areas of the world, particularly in developing countries where hemophilia is treated with fresh frozen plasma or cryoprecipitate. Approximately 80% of individuals using these products are estimated to be HCV infected. Patients with hemophilia thus constitute a high-risk group for acquiring HCV infection [38]. We conclude from this study that despite the reduction in the prevalence of HBV and HCV infection among multitransfused children over the past few years, the prevalence remains high in Upper Egypt. More efforts should be made to ensure complete vaccination of children and provide poster doses if needed. Furthermore, blood screening should be performed using more advanced techniques.
